# Blood pressure in bipolar disorder: evidence of elevated pulse pressure and associations between mean pressure and mood instability

**DOI:** 10.1186/s40345-020-00209-x

**Published:** 2021-02-01

**Authors:** Niall M. McGowan, Molly Nichols, Amy C. Bilderbeck, Guy M. Goodwin, Kate E. A. Saunders

**Affiliations:** 1 Department of Psychiatry, University of Oxford, Warneford Hospital, Oxford, OX3 7JX UK; 2 Academic Centre, John Radcliffe Hospital, Oxford University Clinical School, Oxford, OX3 9DU UK; 3 Oxford Health NHS Foundation Trust, Warneford Hospital, Oxford, OX3 7JX UK; 4 NIHR Oxford Health Biomedical Research Centre, Oxford, OX3 7JX UK

**Keywords:** Blood pressure, Mood instability, Bipolar disorder, Borderline personality disorder, Ecological momentary assessment

## Abstract

**Background:**

Bipolar disorder (BD) is associated with excess and premature cardiovascular mortality. Elevated blood pressure (BP) is a leading contributor to cardiovascular risk. However, few studies have examined BP in BD in comparison to other psychiatric disorders. Furthermore, the association between BP and mood instability is not presently clear despite increasing interest in repurposing existing antihypertensive medications as possible novel BD treatments. Thus we examined BP differences between BD and borderline personality disorder (BPD), a disorder with a similar symptom profile through chronic mood instability.

**Methods:**

A total of 106 adults (38 BD, 25 BPD, and 43 healthy controls), evaluated in the Automated Monitoring of Symptom Severity (AMoSS) study, completed a week-long home blood pressure monitoring assessment and ecological momentary assessment of mood. We examined group-wise differences in mean BP and BP variability and their association with mood instability.

**Results:**

BD individuals had a significantly wider resting pulse pressure (40.8 ± 7.4, mmHg) compared to BPD (35.7 ± 5.3, mmHg, P = 0.03) and control participants (37.3 ± 6.3, mmHg, P = 0.036). Systolic BP was negatively associated with sad mood instability, and all measures of mean BP (systolic, diastolic, and mean arterial pressure) were negatively associated with positive mood instability.

**Conclusions:**

This study demonstrates BP differences between BD and healthy and clinical controls that are within a normotensive range. Early pulse pressure widening may be a modifiable pathophysiological feature of BD that confers later cardiovascular risk. BP may be an important transdiagnostic predictor of mood instability and a potential explicit treatment target.

## Background

Bipolar disorder (BD) is a severe affective disorder associated with substantial physical co-morbidity and excess mortality. Life expectancy for those with BD is reduced by 8–12 years compared to the general population (Laursen 2011; Crump et al. 2013; Kessling et al. 2015a). Cardiovascular disease (CVD) is the leading cause of premature death in BD, accounting for over one-third of deaths in people with the disorder (Osby et al. 2001; Westman et al. 2013). A striking feature of CVD risk in BD is the early age of incidence; CVD occurs up to 17 years earlier in individuals with BD compared to the general population, and those aged younger than 40 years have an eightfold higher risk of CVD mortality (Goldstein et al. 2015; Westman et al. 2013). Several factors may contribute to this elevated risk. Prevalence rates of conditions that confer greater CVD risk such as diabetes and metabolic syndrome are increased in BD compared to the general population (Coello et al. 2019a; Charles et al. 2016). Lifestyle factors such as smoking, alcohol consumption, and physical inactivity, are also more common in BD (Brown et al. 2013). However, accounting for these risk factors does not fully explain excess CVD risk in BD (Hayes et al. 2017). Antipsychotic and mood stabiliser medications are also associated with cardiovascular risk factors but there is no direct association with excess CVD mortality (Osborn et al. 2015). Moreover, elevated CVD mortality had been documented in BD before such drugs were widely used for its treatment (Weiner et al. 2011).

Hypertension, persistent high resting blood pressure (BP), is the leading single contributor to all-cause mortality and disability worldwide (Forouzanfar et al. 2016). It is normally defined by a clinic systolic blood pressure (SBP) > 140 mmHg or diastolic blood pressure (DBP) > 90 mmHg. Importantly, the association between BP and CVD is continuous so that even increased BP in the normotensive range is associated with greater risk (Oparil et al. 2018; Whelton et al. 2020). Several studies have demonstrated greater prevalence of hypertension in BD (Ayerbe et al. 2018). The UK National Institute for Health and Care Excellence (NICE) guidelines recommend hypertension screening for those with BD at the earliest opportunity (NICE 2014). However, psychiatric inpatient service audit reveals inconsistent and sub-optimal BP screening practices in patients with BD (Kelbrick and Abdaldayim 2014). Prevalence of hypertension is known to increase dramatically from middle-age onwards (Pinto et al. 2007; Wolf-Maier et al. 2003), but as a consequence, excess cardiovascular risk may be underappreciated in younger patients with BD (Osby et al. 2016; Kessling et al. 2015b), so measurement of BP assumes great importance.

Home blood pressure monitoring (HBPM) at regular intervals over several days is recommended to confirm clinic detection of hypertension. As a research tool it provides an ecologically valid measure of BP and is easy to monitor. Importantly, as HBPM assesses BP at several time-points it can also quantify blood pressure variability (BPV) over a mid to long-term assessment period (Chadachan et al. 2018). Although the mechanisms producing BPV are incompletely understood it is assumed to arise from the interaction of environmental and behavioural factors with intrinsic actions of the autonomic nervous system and circadian timing system (Parati et al. 2013; Grassi et al. 2012). Several studies have shown that greater BPV is a risk factor for poor cardiovascular outcomes and mortality independent of the effect of mean BP (Stevens et al. 2016). As far as we are aware BPV has not been examined previously in BD.

Elevated BP has been linked to several other psychiatric disorders (Stein et al. 2014). Thus a clinical comparison group with similar symptoms is desirable to draw conclusions specific to BD. Borderline personality disorder (BPD) has a very similar clinical phenotype to BD, involving overlapping symptoms such as abrupt mood lability and impulsivity (Ghaelmi et al. 2014). Accumulating evidence suggests that BPD has an underappreciated hypertensive and CVD risk similar to BD (Roininen et al. 2019). BPD is therefore an interesting comparator group for BD in addition to healthy control subjects.

Mood episode frequency of mania/hypomania and severity of depression appear to be associated with excess cardiovascular mortality (Fiedorowicz et al. 2009, 2014). Little is known about the association between elevated BP as an isolated cardiovascular risk factor and subsyndromal mood symptoms which are common between episodes in BD. Recent evidence also indicates that antihypertensive medications may confer treatment benefits for people with mood disorders. In particular angiotensin antagonists and calcium channel blockers have been suggested as promising candidates for repurposing for future BD treatment (Shaw et al. 2019; Cipriani et al. 2016). The therapeutic mechanisms of each are incompletely understood but hypothesised to involve abnormalities in renin-angiotensin system and altered L-type calcium channel signalling in BD (Barbosa and Ferreira 2020; Cipriani et al. 2016). However, given their primary indication is for BP management, a basic gap in the knowledge exists regarding the association between BP and mood disorder symptoms. Mood instability is characteristic of both BD and particularly BPD (Tsanas et al. 2016) and is a continuous variable, present to varying degrees in the general population and a transdiagnostic feature of several psychiatric conditions (Harrison et al. 2018). Elucidating the association between BP and a core dimension of the BD phenotype such as mood instability is fundamental to developing further the theoretical basis for emerging repurposed treatments.

Here, we examine differences in BP and BPV between participants with BD, BPD and healthy controls (HC) who took part in the Automated Monitoring of Symptom Severity (AMoSS) study. We assess group-wise differences in resting pulse rate (HR), SBP, DBP, and two BP derived measures with superior predictive strength of CVD risk: pulse pressure (PP) and mean arterial pressure (MAP) (Franklin et al. 2009). Based on previous hypertension studies in BD, we hypothesised that BP and BPV would be greater in BD than BPD and HC. Additionally, we performed exploratory analyses to examine the association of BP/BPV measures with BD symptom measures and mood instability, assessed via parallel digital ecological momentary assessment, as a means to expand on the role for explicit antihypertensive treatment potential for mood disorders.

## Methods

### Participants

Participants consisted of 106 individuals recruited to take part in the Automated Monitoring of Symptom Severity (AMoSS) study at the University of Oxford. In total 141 participants were originally recruited, with 113 participants undergoing a week of ‘high-intensity’ behavioural digital monitoring during which participants’ mood and blood pressure were monitored daily. Five participants were excluded due to non-completion of the monitoring phase and a further two were excluded due to technical error or missing data.

Participants were recruited from the community in the case of HC, or from the community, out‐patient services or from study registration lists in the case of BD and BPD participants. Diagnoses were confirmed prior to study admission by psychiatric interview using the Structured Clinical Interview for DSM‐IV and the BPD module of the International Personality Disorders Examination. Individuals with comorbid diagnosis of BD and BPD were excluded from participation. Exclusion criteria for HCs were a history of neurological disorder or head injury, psychiatric diagnosis or having a first degree relative with BD or BPD. Ethical approval for this study was granted by the NRES Committee East of England—Norfolk (13/EE/0288) and informed consent was obtained from all participants taking part.

### Blood pressure

Blood pressure recordings were obtained for each participant over a seven-day HBPM period. Participants used an electronic blood pressure monitor (UA787 Digital Blood Pressure Monitor, A&D Medical) to measure their resting BP. Participants were instructed to take measurements seated upright, wearing the correctly adjusted cuff on their upper left arm, supported, and at heart level (mid-sternal). Participants measured their BP three times each day, once in the morning, afternoon, and evening. At each time-point participants’ recorded three measurements. SBP, DBP and pulse rate were recorded by the device and transferred via Bluetooth to the study smartphone application.

PP (SBP–DBP) and MAP (DBP + 1/3 · PP) were calculated for each time-point offline. The daily mean of each participant’s BP data was used to assess mid-term BPV between days with the grand averages used as inter-individual measures of BP. To overcome the inherent correlation between standard deviation and mean BP values we used the coefficient of variation (CV) expressed as percentage as a measure of day-to-day BPV.

### Questionnaire assessments

The Quick Inventory of Depressive Symptomatology—Self Report (QIDS) is a 16 item scale that reflects 9 constituent components of depression that participants report on over the previous week. Total QIDS scores range from 0 to 27. The suggested clinical ranges are 5 or less denoting normal, 6–10 denoting mild depression, 11–15 denoting moderate depression, 16–20 denoting severe depression, and 21–27 denoting very severe depression (Rush et al. 2003).

Altman Self Rating Mania Scale (ASRM) is a five-item scale examining severity of manic symptoms arranged across domains of elevated mood, self-confidence, sleep disturbance, speech patterns and amount, and activity level over the previous week. Items are scored on a 0 (symptom-free) to 4 (present nearly all the time) scale, with total scores ranging from 0 to 20. A score of 6 or higher is indicative of mania, or hypomania, with symptom severity indicated by increasing scores (Altman et al. 1997).

Participants completed both questionnaires at the end of the HBPM week period using the True Colours remote monitoring mood system (www.truecolours.nhs.uk).

### Mood Zoom

Mood Zoom (MZ) is a 6-item questionnaire that was designed as part of the AMoSS study to enable a compact ecological momentary assessment of mood instability using participants’ smartphone devices. MZ consists of six mood items arranged in three factors: negative (‘anxious’, ‘sad’), positive (‘elated’, ‘energetic’), and irritable (‘irritable’, ‘angry’). Previous work has demonstrated strong correlation between MZ components and standardised measures of depression, anxiety and mental health (Tsanas et al. 2016). Parallel to the HBPM period during the ‘high intensity’ study week, participants were asked to rate 10-times each day to what extent each of the six mood items reflected their current mood on a 7‐point Likert scale ranging from ‘Not at all’ to ‘Very much. Digital prompts for participants to provide their mood rating were delivered at equally spaced intervals throughout each day, between 1000 and 2000 h (i.e. approx. every hour).

To quantify mood instability for each of the six MZ items we used the root mean square of successive differences (RMSSD) over the week of mood monitoring. The RMSSD is a measure of variability reflecting both the temporal order and amplitude of the data (Ebner-Priemer et al. 2009) and is a commonly used measure of mood instability in studies using ecological momentary assessment. Mood diary entries with missing data were excluded pair-wise and the next adjacent case was used for the calculation of RMSSD.

### Data analysis

Normal distribution of data was assessed using Shapiro–Wilk tests of normality and visual inspection of Q-Q plots. BP and BPV variables were normally disturbed but questionnaire and MZ RMSSD values were skewed. Sample descriptive data were analysed using chi-square tests of independence for categorical data and one-way ANOVAs (mean ± SD) or Kruskal–Wallis non-parametric tests (median ± IQR) for continuous data that were normally distributed or non-normally distributed, respectively. Group-wise comparisons of BP measures were analysed using ANCOVAs with participant age and BMI inserted as covariates. All post-hoc tests were conducted using Bonferroni correction for multiple comparisons. Exploratory correlations between questionnaire/mood outcomes and BP/BPV variables were performed using Spearman’s rank coefficient with the Benjamini–Hochberg false discovery rate applied to P-values. Adjusted P-values less than 0.05 were considered statistically significant. All analyses were conducting using SPSS v25 (IBM) and R v3.6.3 (R core team).

## Results

### Demographic characteristics

Demographic characteristics of participants are shown in Table 1. After exclusion of five participants for incompletion or technical errors, the final sample analysed consisted of 38 participants with BD (63% female), 25 participants with BPD (88% female), and 43 HC (67% female), aged between 18 and 64 years. There was a greater number of female participants among the BPD group compared with BD and HC groups. Groups were well matched for age and ~ 80% of the sample was younger than 50 years. The mean BMI of clinical groups was only slightly higher than controls. The proportion of smokers was greater in both clinical groups compared to HCs. Groups did not differ by the proportion of alcohol drinkers.

**Table 1:** Demographic and clinical characteristics of sample

	BD (*n* = 38)	BPD (*n* = 25)	HC (*n* = 43)	Test statistic	*P*	*Post-hoc*
Gender						
Female n (%)	24 (63.2)	22 (88)	29 (67.4)	χ^2^ = 4.881	0.087	–
Age, year ± SD	39.0 ± 13.1	34.1 ± 11.2	39.84 ± 12.9	*F* = 1.794	0.171	–
BMI, kg/m^2^ ± SD	26.8 ± 4.1	27.4 ± 6.3	24.5 ± 4.3	*F* = 3.657	*0.029*	n.s
Smoker, n (%)	7 (21.9)	7 (30.4))	2 (5)	χ^2^ = 7.818	*0.022*	BD > HC BPD > HC
Drinks Alcohol, n (%)	28 (77.8)	14 (58.3)	31 (72)	χ^2^ = 2.691	0.260	–
Questionnaire assessment						
QIDS, mdn ± IQR	5.5 ± 6.5	12 ± 12.5	2 ± 1.5	χ^2^ = 50.586	< *0.001*	BD > HC BPD > HC BPD > BD
ASRM, mdn ± IQR	1 ± 2	1 ± 3.5	0 ± 0	χ^2^ = 11.663	< *0.003*	BD > HC BPD > HC
Mood Zoom Items						
anx RMSSD ± IQR	0.87 ± 0.54	1.39 ± 0.58	0.55 ± 0.65	χ^2^ = 42.81	< *0.001*	BD > HC BPD > HC BPD > BD
sad RMSSD ± IQR	0.72 ± 0.71	1.40 ± 0.43	0.31 ± 0.75	χ^2^ = 41.662	< *0.001*	BD > HC BPD > HC BPD > BD
elt RMSSD ± IQR	0.67 ± 0.73	1.14 ± 1.06	0.67 ± 0.88	χ^2^ = 10.489	*0.005*	BPD > HC
enr RMSSD ± IQR	0.93 ± 0.70	1.22 ± 0.90	0.79 ± 0.51	χ^2^ = 11.558	*0.003*	BPD > HC BPD > BD
ang RMSSD ± IQR	0.71 ± 0.58	1.24 ± 0.97	0.31 ± 0.49	χ^2^ = 36.695	< *0.001*	BD > HC BPD > HC BPD > BD
irr RMSSD ± IQR	0.91 ± 0.71	1.53 ± 1.05	0.48 ± 0.78	χ^2^ = 39.167	< *0.001*	BD > HC BPD > HC BPD > BD
Medication						
Using psychotropic medication, n (%)	36 (94.7)	19 (87.3)	–	χ^2^ = 4.775	*0.029*	–
Lithium	16 (42.1)	0 (0)	–	χ^2^ = 14.11	< *0.001*	–
Anticonvulsant	15 (39.5)	1 (4)	–	χ^2^ = 10.015	*0.002*	–
Antipsychotic	25 (65.8)	5 (20)	–	χ^2^ = 12.675	< *0.001*	–
Antidepressant	13 (34.2)	19 (76)	–	χ^2^ = 10.536	*0.001*	–
Hypnotic	3 (7.9)	1 (1.5)	–	χ^2^ = 0.339	0.561	–
Anxiolytic	1 (2.6)	6 (24)	–	χ^2^ = 6.971	*0.008*	–

F test statistic denotes univariate ANOVA result; χ^2^ test statistic denotes chi-square test of independence (for categorical data) or Kruskal–Wallis non-parametric test (comparing the medians of continuous variable). *IQR*  interquartile range, *mdn* median, *SD* standard deviation

Symptom assessments in this sub-sample of participants were congruent with previously reported findings of the full AMoSS study: depressive symptomatology was significantly greater in both clinical groups compared to HC, and in BPD compared to BD. Manic symptoms were greater in clinical groups compared to HC. Mood instability characterised by MZ assessment revealed greater variability for anxious and sad items in both clinical groups compared to HC; greater variability of elated mood in BPD compared to HC, and greater variability of energetic mood in BPD compared with both HC and BD; for anger and irritability, both clinical groups were significantly more variable than HC, and BPD was significantly more variable compared to BD.

Psychotropic medication use differed between BD and BPD with a larger proportion of BD participants taking medication. Lithium use was exclusive to the BD group, anticonvulsant and antipsychotic use was more common in BD compared to BPD. Antidepressant and anxiolytic use was more common in BPD compared to BD. Four of the BPD group had prescriptions for propranolol to take as required for anxiety: since their use was uncertain, patients are included in the analysis.

### Blood pressure

Out of 106 participants monitored, 91 (86%) were normotensive, and 15 (14%) were hypertensive (2 SBP/DBP hypertension, 1 isolated systolic hypertension, 12 isolated diastolic hypertension based on the 2018 ESH/ESC guideline definitions for HBPM hypertension; ≥ 135 mmHg SBP and/or ≥ 85 mmHg DBP) (Williams et al. 2018).

**Fig. 1:**
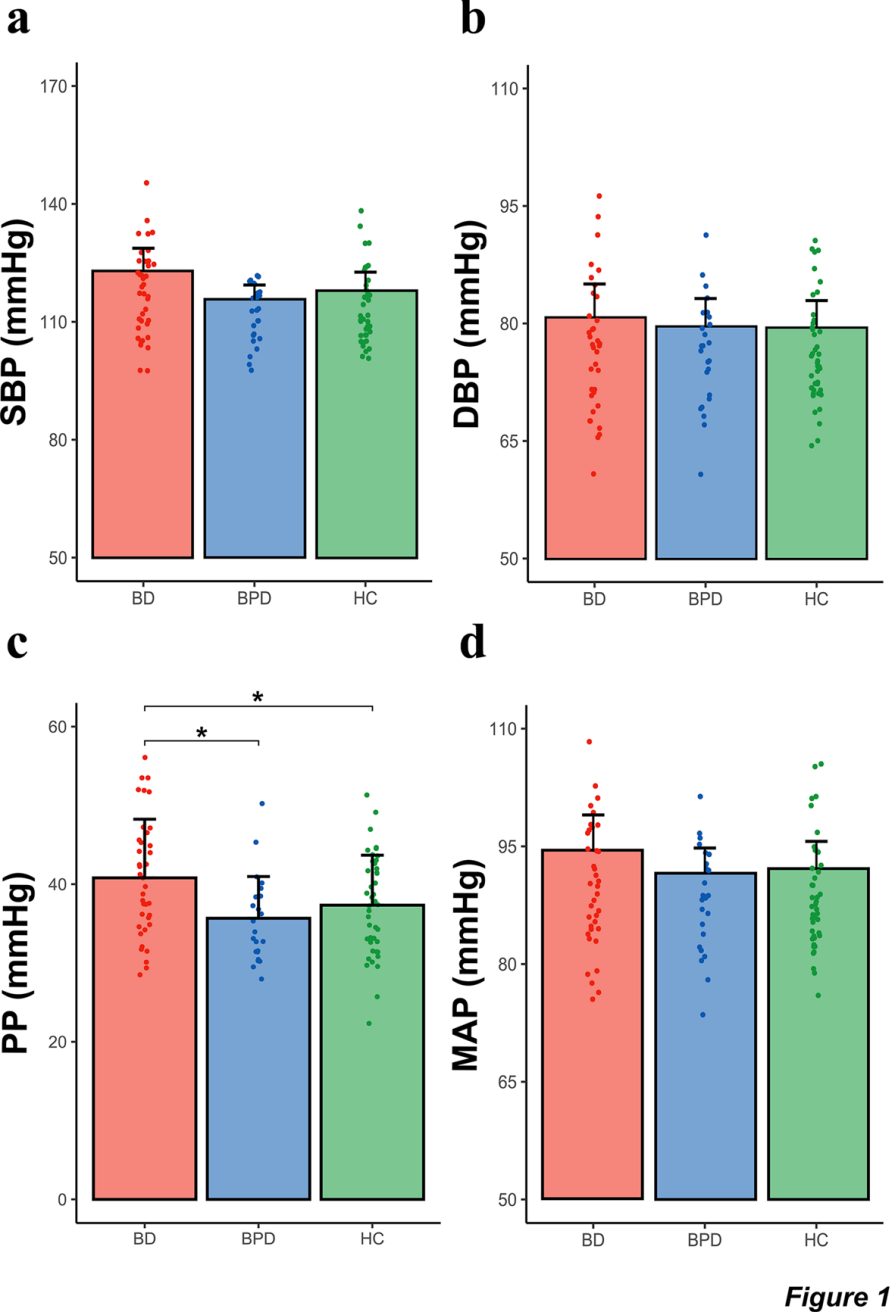
Group-wise comparison of mean blood pressure variables. Bars represent group means with error bars indicating standard deviation. Variables plotted are as follows **a** systolic blood pressure (SBP); **b** diastolic blood pressure (DBP); **c** pulse pressure (PP); **d** mean arterial pressure (MAP). All comparisons control for age and BMI as covariates with Bonferroni post-hoc correction applied. *denotes P < 0.05

Expectedly, BP measures were significantly positively associated with age and BMI (Additional file 1: Table S1), thus both variables were retained as covariates in ANCOVA analyses. Group-wise comparisons did not show a significant effect of diagnosis on mean HR, SBP or DBP (Table 2, Fig. 1a, b). The effect of diagnosis on PP was significant, *F*(1, 100) = 4.71, P = 0.011, $\eta_{P}^{2}$ = 0.085. Post-hoc comparisons revealed that BD had significantly higher PP values (40.8 ± 7.4, mmHg) compared to BPD (35.7 ± 5.3, mmHg, P = 0.03) and HC (37.3 ± 6.3, mmHg, P = 0.036) (Table 2, Fig. 1c). There was no significant effect of diagnosis on MAP (Table 2, Fig. 1d) or BPV estimated by the day-to-day coefficient of variation (Table 2, Additional file 1: Figure S1).

**Table 2:** Group-wise comparisons of BP and BPV measures

Variable	BD (*n* = 38)	BPD (*n* = 25)	HC (*n* = 43)	ANCOVA		
				*F*_*(2, 100)*_	*P*	$\eta_{P}^{2}$
*BP metric*						
HR, BPM ± SD	69.3 ± 10.8	75 ± 8.9	67.57 ± 8.8	2.576	0.081	0.049
SBP, mmHg ± SD	117.7 ± 11	112 ± 7.3	113.6 ± 9	2.671	0.074	0.050
DBP, mmHg ± SD	76.9 ± 8.1	76.4 ± 6.8	76.3 ± 6.6	0.126	0.881	0.002
PP, mmHg ± SD	*40.8 ± 7.4*	*35.7 ± 5.3*	*37.3 ± 6.3*	*4.711*	*0.011*	*0.085*
MAP, mmHg ± SD	90.5 ± 8.5	88.3 ± 6.5	88.7 ± 6.9	0.494	0.612	0.010
*BPV metric*						
cvHR, % ± SD	10.6 ± 3.6	10.5 ± 3.5	10.5 ± 3.3	0.056	0.945	0.001
cvSBP, % ± SD	6.2 ± 1.7	6.5 ± 2.2	6.4 ± 2	1.537	0.220	0.030
cvDBP, % ± SD	7.2 ± 2.3	7.9 ± 2.3	7.8 ± 2.2	1.427	0.245	0.028
cvPP, % ± SD	13.6 ± 3.7	14.1 ± 4	14.6 ± 5.3	0.586	0.559	0.012
cvMAP, % ± SD	6.2 ± 1.9	6.8 ± 2.2	6.6 ± 1.9	2.458	0.091	0.047

ANCOVA results are reported with age and BMI inserted as covariates

Exploratory group-wise assessments and sensitivity analyses were conducted to determine the effects of potential confounding influences on BP. Alcohol use was associated with significantly wider PP (Additional file 1: Table S2) and smoking with significantly higher pulse-rate (Additional file 1: Table S3). However, controlling for each as additional covariates did not explain the effect of diagnosis on PP (Additional file 1: Tables S4 and S5), and when controlling for smoking status significant between group differences in SBP emerged (Additional file 1: Table S5; Bonferroni post-hoc BD > BPD, P = 0.028). Neither lithium nor anticonvulsant use was associated with BP differences among BD participants (Additional file 1: Tables S6 and S7). Among BD and BPD, antipsychotic use was associated with higher SBP (Additional file 1: Table S8) and antidepressant use with lower PP (Additional file 1: Table S9), partly reflecting the differing medication rates between both diagnoses. Hypnotic and anxiolytic users numbered too few to compare statistically. Thus, we conducted a sensitivity analysis on the differences detected between BD, BPD and HC, while additionally controlling for any psychotropic medication use and found the effect of diagnosis on PP was preserved (P = 0.043; Additional file 1: Table S10). There was insufficient representation of males among the BPD group to adequately include gender as a covariate but a sub-group sensitivity analysis between BD and HC that additionally controlled for gender revealed the same pattern of greater PP in BD (P = 0.029; Additional file 1: Table S11).

### Association with mood instability

**Fig. 2:**
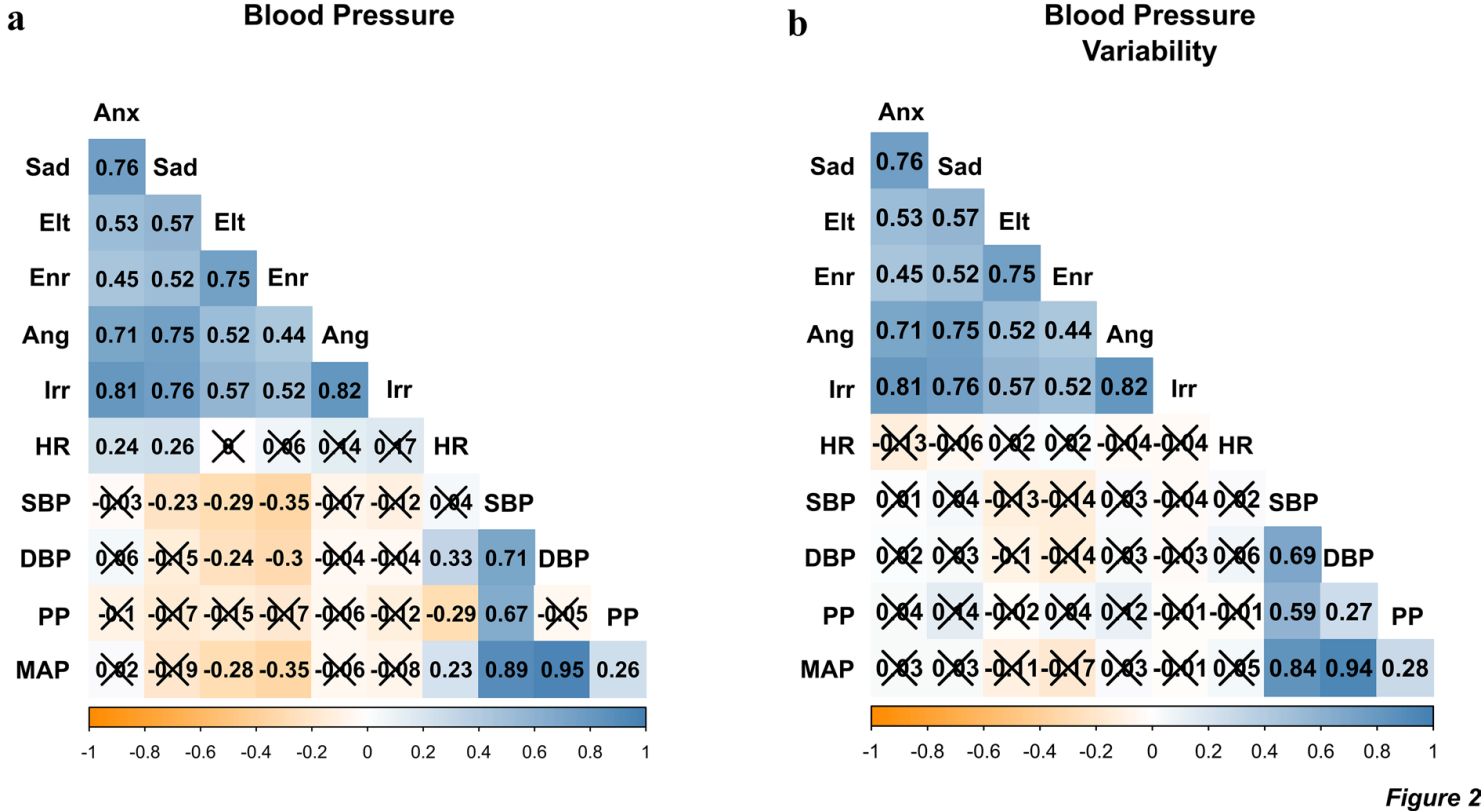
Correlations between blood pressure and mood instability. Correlation plot shows Spearman rank-order correlation matrix of mood instability variables from MZ digital questionnaire and **a** mean BP variables and **b** BPV variables measured via the coefficient of variation. Mood instability of each MZ item is estimated by the RMSSD of mood ratings. Strength and direction of correlation is indicated by colour legend. Abbreviations are as follows: Anx = anxious, Elt = elated; Enr = energetic; Ang = angry; Irr = irritable; HR = pulse rate; SBP = systolic blood pressure; DBP = diastolic blood pressure; PP = pulse pressure; MAP = mean arterial pressure. Crossed-out coefficients represent non-significant correlations (P > 0.05, FDR correction applied)

Associations between BP mean values and MZ items (RMSSD) are shown in Fig. 2a. SBP was negatively associated with instability on the sad mood item and the positive mood items, elated and energetic (higher SBP, lower mood instability). Positive mood items were also negatively associated with DBP, and MAP (higher BP metric, lower mood instability). No significant associations were detected between BP values and angry and irritable mood items, and BPV measures (CV of each BP measure) were not correlated with any MZ items (Fig. 2b). Higher resting heart rate correlated with greater instability of negative mood items, anxiety and sad. There was no association between mean BP and BPV metrics and QIDS and ASRM scores (Additional file 1: Figure S2a, b). The inverse correlation between mean BP and negative/positive mood instability was similar for all groups in direction and magnitude (Additional file 1: Figure S3).

## Discussion

Participants with BD exhibited significantly wider PP than HCs and participants with BPD but groups did not differ in terms of resting measures of HR, SBP, DBP or MAP. BP measures and mood instability measures were inversely related. Higher resting HR was associated with greater mood instability on negative MZ items.

### Blood pressure

In most adults both SBP and DBP rise continuously from the age of 30 years. This linear trend continues into old-age for SBP whereas DBP typically plateaus during the fifth decade of life (Franklin et al. 1997). Consequently, PP (SBP–DBP) widens markedly during middle-age. Additionally, BP increases with higher BMI (Kang et al. 2017). Our findings, corrected for age and BMI, suggest a premature elevation of PP in BD.

Increased PP denotes an increase in arterial stiffness and is a correlate of several markers of sub-clinical CVD (Winston et al. 2013). It is an important predictor of CVD risk and cardiovascular mortality, with evidence supporting a stronger association with PP in normotensive individuals compared to those with hypertension (Hadaegh et al. 2012; Benetos et al. 1998; Benetos et al. 1997; Blacher et al. 2000). Additionally, PP may have superior predictive ability for certain cardiovascular outcomes. Data from the Framingham Heart Study suggest that PP is a better predictor of coronary heart disease than SBP and DBP (Franklin et al. 1999). Similar findings have been described for congestive heart failure and myocardial infarction in individuals aged > 65 years (Chae et al. 1999; Vaccarino et al. 2000). In a primarily normotensive cohort such as the current sample, elevated PP in BD may indicate greater sub-clinical risk of adverse cardiovascular outcome compared to healthy and clinical controls. The use of dopamine antagonist drugs and lithium was more common in BD than BPD. Elevated risk for hypertension has been described with antipsychotic use in BD but weight gain may be the main mediator (Correll et al. 2015; Vancampfort et al. 2015) and BMI was controlled in the present comparison. Over 40% of BD participants in this study used lithium. Lithium has been described in one small sample-size study to elevate blood pressure when used as monotherapy (Johnstone et al. 1990), but we did not detect differences between lithium users and non-users. Future studies with greater power are necessary to interrogate further the effects of psychotropic medication and polypharmacy on BP risk and how it relates to BD.

Previous work has focused on the association between BD and hypertension (Johannessen et al. 2006; Goldstein et al. 2009; Chien et al. 2013). Our findings highlight the importance of considering BP as a continuous variable and examining sub-clinical BP differences. This is particularly important given that CVD risk is also related to higher BP well within the normotensive range (Whelton et al. 2020) and excess CVD mortality is described in BD before the age where hypertension is normally screened (Osby et al. 2016; Kessling et al. 2015b).

CVD risk is not unique to BD among the psychiatric disorders (Foguet-Boreu et al. 2016). The reasons for this association are multifactorial and include elevated blood pressure, which has been described across several psychiatric disorders (Stein et al. 2014). However, BPD failed to show any differences compared to control participants. Recent evidence suggests a genetic component to CVD in BD, with a higher prevalence of cardiovascular risk factors and increased Framingham risk score present among unaffected siblings/first-degree relatives of individuals with BD (Tsao et al. 2019; Coello et al. 2019b). Furthermore, several overlapping pleiotropic genes have been implicated with both CVD and BD (Amare et al. 2017). The heritability of BPD is probably lower than BD (Skoglund et al. 2019). Dysfunctional behaviour may be more likely to mediate the association of BPD with CVD risk (Grove et al. 2017; Moran et al. 2007). Therefore, treatment effects of recommended psychological therapies that remodel behaviour and improve interpersonal functioning may reduce the risk in BPD.

### Mood instability

Mood instability findings in this sub-sample of the AMoSS study were consistent with the full sample (Tsanas et al. 2016). Taken transdiagnostically, our findings reveal interesting associations with HBPM variables. Mean SBP was negatively correlated with instability of MZ item sad, and all measures of mean BP (SBP, DBP, and MAP) were negatively associated with instability of positive MZ items elated and energetic. The direction of these associations is unexpected. The hypothesis that emotional distress and elevated BP are positively linked originates from over a century ago (Hildrum et al. 2008). However, few studies have demonstrated prospective evidence to support it; anger shows the strongest association with limited support for depression and anxiety (Rutledge and Hogan 2002). Conversely, several studies report an association between lower BP and depression and anxiety (Hildrum et al. 2008, 2011; Speerforck et al. 2019). That BP was inversely associated with instability of sadness in the current sample is consistent with these trends. However, we did not observe any association with instability among anger or irritability items. An association between emotional reactivity in remitted BD and elevated SBP/DBP has been described previously (Dargel et al. 2018). The authors suggested that BP differences may discriminate BD individuals with high inter-episode mood instability. However, the present findings suggest strongest associations with lower instability among energetic and elated mood components, respectively. Several aspects of our assessment of mood might account for different findings. Our experience sampling methods reflect mood changes in daily life, whereas previous studies have used retrospective self-report instruments often reporting psychological traits rather than current mood per se*.* Notably, we did not detect any association between BP and manic symptoms. Thus the inverse association between mean BP and mood instability for energetic and elated items may emerge from causes other than subsyndromal mood elevation (e.g. low mean activity and consequently more stable energy ratings for individuals with high BP). Furthermore, only the BPD group showed significant mood instability on these items and thus the generalisability of BP findings and association with more severe mood alterations in remitted BD is unclear. Higher resting HR was associated with greater mood instability of negative MZ items. A higher resting HR (and lower inter-beat-interval heart rate variability) is consistent with lower parasympathetic nervous system activity, which in turn is associated with greater mood instability and difficulties in emotion regulation (Koval et al. 2013; Williams et al. 2015).

The current findings are cross-sectional and thus any potential causal association between BP and mood instability is unclear. However, the associations we describe between BP and mood have implications for future predictive modelling studies and the search for intervention targets. Prospective longitudinal monitoring of BP changes and mood may facilitate the use of this biomarker for identifying individuals at increased risk of residual mood symptoms and episode recurrence. Furthermore, the integration of contemporaneous BP monitoring within antihypertensive experimental medicine studies may clarify their therapeutic role as proposed for BD. Large-scale cohort data suggest that some antihypertensives (e.g. angiotensin antagonists) may be associated with decreased risk of mood disorders, while others (e.g. beta-blockers) are associated with increased risk (Shaw et al. 2019; Boal et al. 2016). Given the associations between BP and mood described here, future healthcare record studies and experimental studies addressing antihypertensive drugs should examine the mediating influence of BP on mood in order to disentangle potential therapeutic mechanism behind their purported effects on mood.

### Blood pressure variability

We did not detect any differences for BPV between groups or any meaningful associations with mood instability. The study demonstrated good tolerability of HBPM monitoring in BD and BPD. Importantly, we assessed mid-term BPV and thus our observations are interpreted within the context of day-to-day changes, limited to waking hours only. Short-term BPV can be measured through ambulatory blood pressure monitoring (ABPM) and better captures BPV over the course of the 24-h day in 15–30 min intervals (Chadachan et al. 2018). Future studies that employ higher frequency BPV sampling methods such as via ABPM are required to thoroughly assess normal circadian fluctuation and finer scale autonomic oscillations of BP in psychiatric groups. This is particularly relevant in the context of previous findings of differential circadian function and autonomic regulation and their influence upon mood instability in BD and BPD (Carr et al. 2018; McGowan et al. 2019, 2020).

### Limitations

There was a preponderance of female participants in the BPD group compared to BD and HC, consistent with its greater prevalence in women (APA 2013). Importantly, age by sex BP differences have been described previously (Reckelhoff 2001); BP is normally higher in men than women until menopause, after which this trend reverses. However, our analyses controlled for age and elevated PP in BD was observed versus a gender equivalent healthy control group and also with gender entered as a covariate. However, future longitudinal studies are required to understand how age and sex interactions may be different among clinical groups (for example post-menopause in women). Although we controlled for psychotropic drug use in our sensitivity analysis, other limitations are differing medication across the clinical samples and their potential interacting influence via polypharmacy. The current sample sizes are too small to fully examine this effect. Future longitudinal studies that harness digital health records will be informative for understanding the long-term effects of medication on BP in mood disorders. However, a clear strength of our approach was monitoring of BP over multiple days to quantify its variability, which was feasible due to our sample size. HBPM was performed at multiple daily time-points in line with ESH/ESC guidelines but its duration/frequency is limited by participant effort. Studies that use ABPM would have the advantage of better capturing BPV and relating this to intra-daily mood changes. Finally, as described above, our data are cross-sectional and thus we cannot determine a cause-effect relation between BP and mood instability or vice versa.

## Conclusions

The results of a week-long HBPM assessment show an elevated resting pulse pressure in BD relative to BPD and HCs while BPV measures did not differ between groups. Mean BP metrics and resting HR correlated with mood instability monitored via ecological momentary assessment. Together, the results demonstrate subtle BP differences in BD subjects within a normotensive range and that mean BP is associated with mood instability in a transdiagnostic group. Despite the high prevalence of cardiovascular mortality in BD, a focus on prevention and risk management in BD is regrettably lacking (Goldstein et al. 2020). Yet, BP is a highly modifiable risk factor. Our findings identify pulse pressure as potential target for risk management and that BP may be associated with mood instability.

## Supplementary Information

**Additional file 1.** Supplementary Tables and Figures.

## Data Availability

The datasets used and/or analysed during the current study are available from the corresponding author on reasonable request.

## Abbreviations

ABPM: Ambulatory Blood Pressure Monitoring
AMoSS: Automated Monitoring of Symptom Severity
APA: American Psychiatric Association
ASRM: Altman Self-Rating Mania Scale
BD: Bipolar Disorder
BP: Blood Pressure
BPD: Borderline Personality Disorder
BPV: Blood Pressure Variability
CV: Coefficient of Variation
CVD: Cardiovascular Disease
DBP: Diastolic Blood Pressure
ESH/ESC: European Society of Cardiology (ESC) and the European Society of Hypertension (ESH)
HBPM: Home Blood Pressure Monitoring
HC: Healthy Controls
MAP: Mean Arterial Pressure
MZ: Mood Zoom
NICE: National Institute for Health and Care Excellence
PP: Pulse Pressure
QIDS: Quick Inventory of Depressive Symptomatology (Self-Report)
RMSSD: Root Mean Square of Successive Differences
SBP: Systolic Blood Pressure
