# Supplementary material for: Blood pressure in bipolar disorder: evidence of elevated pulse pressure and associations between mean pressure and mood instability
Source: Int J Bipolar Disord. 2021 Feb 1;9:5. doi: 10.1186/s40345-020-00209-x (PMC7847910; doi:10.1186/s40345-020-00209-x)
Supplement: Supplementary file 1 — Additional file 1. Supplementary Tables and Figures. [file 40345_2020_209_MOESM1_ESM.pdf]

SUPPLEMENTARY MATERIAL

Blood pressure in bipolar disorder: evidence of elevated pulse pressure and associations between mean pressure and mood instability

Niall M. McGowan[1]*, Molly Nichols[2], Amy C. Bilderbeck[1], Guy M. Goodwin[1,3], and Kate E.A. Saunders[1,3,4]

Author affiliations:

1 Department of Psychiatry, University of Oxford, Oxford, OX3 7JX, UK

2 Oxford University Clinical School, Academic Centre, John Radcliffe Hospital, Oxford, OX3 9DU, UK

3 Oxford Health NHS Foundation Trust, Warneford Hospital, Oxford, OX3 7JX, UK

4 NIHR Oxford Health Biomedical Research Centre, Oxford, OX3 7JX, UK

***Corresponding Author: Dr Niall M. McGowan, Department of Psychiatry, University of Oxford, Warneford Hospital, Oxford, OX3 7JX, UK.**

Corresponding Author Email: niall.mcgowan@psych.ox.ac.uk

Supplementary Table 1: Correlation matrix of age, BMI, and blood pressure variables

	Age	BMI	HR	SBP	DBP	PP
**BMI**	0.077	—				
**HR**	**-0.244 ***	**0.213 ***	—			
**SBP**	**0.471 *****	**0.343 *****	0.041	—		
**DBP**	**0.267 ****	**0.391 *****	**0.325 *****	**0.709 *****	—	
**PP**	**0.384 *****	0.073	**-0.285 ****	**0.668 *****	-0.052	—
**MAP**	**0.376 *****	**0.402 *****	**0.227 ***	**0.890 *****	**0.952 *****	**0.256 ****

Correlations are with Pearson product moment coefficient. * P<0.05; ** P<0.01; *** P<0.001

Supplementary Table 2: Group-wise comparison of alcohol use on blood pressure variables

Variable	Alcohol Drinkers (*n* = 73)	Alcohol Non-drinkers (*n* = 30)	Student's t-test	
			T	*P*
**BP Metric**				
HR, BPM ± SD	68.7 ± 8.8	72.6 ± 12.1	1.602†	0.117
SBP, mmHg ± SD	115.6 ± 10.2	112.2 ± 7.8	1.616	0.109
DBP, mmHg ± SD	76.6 ± 6.9	76.4 ± 8.2	0.134	0.894
PP, mmHg ± SD	**38.9 ± 6.9**	**35.8 ± 5.5**	**2.219**	**0.029***
MAP, mmHg ± SD	89.6 ± 7.5	88.3 ± 7.7	0.788	0.433

†Inequality of variances; Welch's t-test used. *P<0.05

Supplementary Table 3: Group-wise comparison of smoking on blood pressure variables

Variable	Smokers (*n* = 16)	Non-smokers (*n* = 79)	Student's t-test	
			T	*P*
**BP Metric**				
HR, BPM ± SD	**77.6 ± 11.5**	**68.2 ± 9.1**	**3.618**	**<0.001*****
SBP, mmHg ± SD	116.3 ± 10.9	114.4 ± 9.5	0.689	0.487
DBP, mmHg ± SD	78.7 ± 9.7	76.3 ± 6.4	1.261	0.210
PP, mmHg ± SD	37.6 ± 5.5	38.1 ± 7.0	0.315	0.753
MAP, mmHg ± SD	91.3 ± 9.8	89.0 ± 6.8	1.120	0.266

***P<0.001

Supplementary Table 4: Sensitivity analysis ANCOVA for alcohol use

Variable	ANCOVA		
**Covariates inserted: Age, BMI, Alcohol use (Yes/No)**	*F*	*P*	η_p^2
**BP Metric**			
HR, BPM ± SD	2.218	0.114	0.044
SBP, mmHg ± SD	2.270	0.109	0.045
DBP, mmHg ± SD	0.205	0.815	0.004
PP, mmHg ± SD	**3.389**	**0.038***	**0.065**
MAP, mmHg ± SD	0.596	0.553	0.012

*P<0.05

Supplementary Table 5: Sensitivity analysis ANCOVA for smoking

Variable	ANCOVA		
**Covariates inserted: Age, BMI, Smoker (Yes/No)**	*F*	*P*	η_p^2
**BP Metric**			
HR, BPM ± SD	2.116	0.126	0.042
SBP, mmHg ± SD	**3.864**	**0.025***	**0.080**
DBP, mmHg ± SD	0.689	0.505	0.015
PP, mmHg ± SD	**3.914**	**0.023***	**0.081**
MAP, mmHg ± SD	1.164	0.200	0.036

*P<0.05

Supplementary Table 6: Group-wise comparison of BD lithium users vs. non-users on blood pressure variables

Variable	Li (*n* = 16)	Non-Li (*n* = 22)	Student's t-test	
			T	*P*
**BP Metric**				
HR, BPM ± SD	67.7 ± 12.1	70.4 ± 12.1	0.748	0.459
SBP, mmHg ± SD	118.3 ± 12.8	117.3 ± 9.8	0.273	0.787
DBP, mmHg ± SD	76.8 ± 9.13	76.9 ± 7.5	0.045	0.964
PP, mmHg ± SD	41.4 ± 7.7	40.3 ± 7.4	0.453	0.653
MAP, mmHg ± SD	90.7 ± 9.8	90.4 ± 7.6	0.089	0.930

Supplementary Table 7: Group-wise comparison of BD anticonvulsant users vs. non-users on blood pressure variables

Variable	Anticon (*n* = 15)	non-Anticon (*n* = 32)	Student's t-test	
			T	*P*
**BP Metric**				
HR, BPM ± SD	70.6 ± 11.2	68.4 ± 10.9	0.587	0.561
SBP, mmHg ± SD	119.5 ± 12.3	116.6 ± 10.2	0.762	0.433
DBP, mmHg ± SD	78.2 ± 8.4	76.1 ± 8.0	0.783	0.439
PP, mmHg ± SD	41.3 ± 7.8	40.5 ± 7.4	0.314	0.755
MAP, mmHg ± SD	91.9 ± 9.2	89.6 ± 8.1	0.843	0.405

Supplementary Table 8: Group-wise comparison of antipsychotic users vs. non-users on blood pressure variables among clinical groups (BD and BPD)

Variable	Antipsyc (*n* = 30)	non-Antipsyc (*n* = 33)	Student's t-test	
			T	*P*
**BP Metric**				
HR, BPM ± SD	72.1 ± 11.9	71.1 ± 9.1	0.372	0.711
SBP, mmHg ± SD	**118.4 ± 10.6**	**112.7 ± 8.7**	**2.328**	**0.023***
DBP, mmHg ± SD	78.1 ± 8.3	75.4 ± 6.7	1.386	0.171
PP, mmHg ± SD	40.4 ± 7.9	37.3 ± 5.9	1.718†	0.087
MAP, mmHg ± SD	91.5 ± 8.3	87.9 ± 6.9	1.896	0.063

†Inequality of variances; Welch's t-test used. *P<0.05

Supplementary Table 9: Group-wise comparison of antidepressant users vs. non-users on blood pressure variables among clinical groups (BD and BPD)

Variable	Antidepr (*n* = 32)	non-Antidepr (*n* = 31)	Student's t-test	
			T	*P*
**BP Metric**				
HR, BPM ± SD	73.9 ± 9.2	69.1 ± 11.2	1.905	0.061
SBP, mmHg ± SD	113.5 ± 7.4	117.5 ± 12.0	1.577†	0.121
DBP, mmHg ± SD	76.7 ± 6.5	76.7 ± 8.7	0.035	0.972
PP, mmHg ± SD	**36.8 ± 6.1**	**40.8 ± 7.6**	**2.341**	**0.023***
MAP, mmHg ± SD	88.9 ± 6.2	90.3 ± 9.3	0.644†	0.520

†Inequality of variances; Welch's t-test used. *P<0.05

Supplementary Table 10: Sensitivity analysis ANCOVA for any psychotropic drug use

Variable	ANCOVA		
**Covariates inserted: Age, BMI, Psychotropic drug use**	*F*	*P*	η_p^2
**BP Metric**			
HR, BPM ± SD	2.170	0.120	0.042
SBP, mmHg ± SD	1.938	0.149	0.037
DBP, mmHg ± SD	0.371	0.691	0.007
PP, mmHg ± SD	**3.237**	**0.043***	**0.061**
MAP, mmHg ± SD	0.569	0.568	0.011

*P<0.05

Supplementary Table 11: Sensitivity analysis ANCOVA for gender (BD vs. HC)

Variable	ANCOVA		
**Covariates inserted: Age, BMI, Gender (M/F)**	*F*	*P*	η_p^2
**BP Metric**			
HR, BPM ± SD	0.110	0.742	0.001
SBP, mmHg ± SD	2.112	0.150	0.027
DBP, mmHg ± SD	0.051	0.822	0.001
PP, mmHg ± SD	**4.956**	**0.029***	**0.061**
MAP, mmHg ± SD	0.207	0.650	0.003

*P<0.05

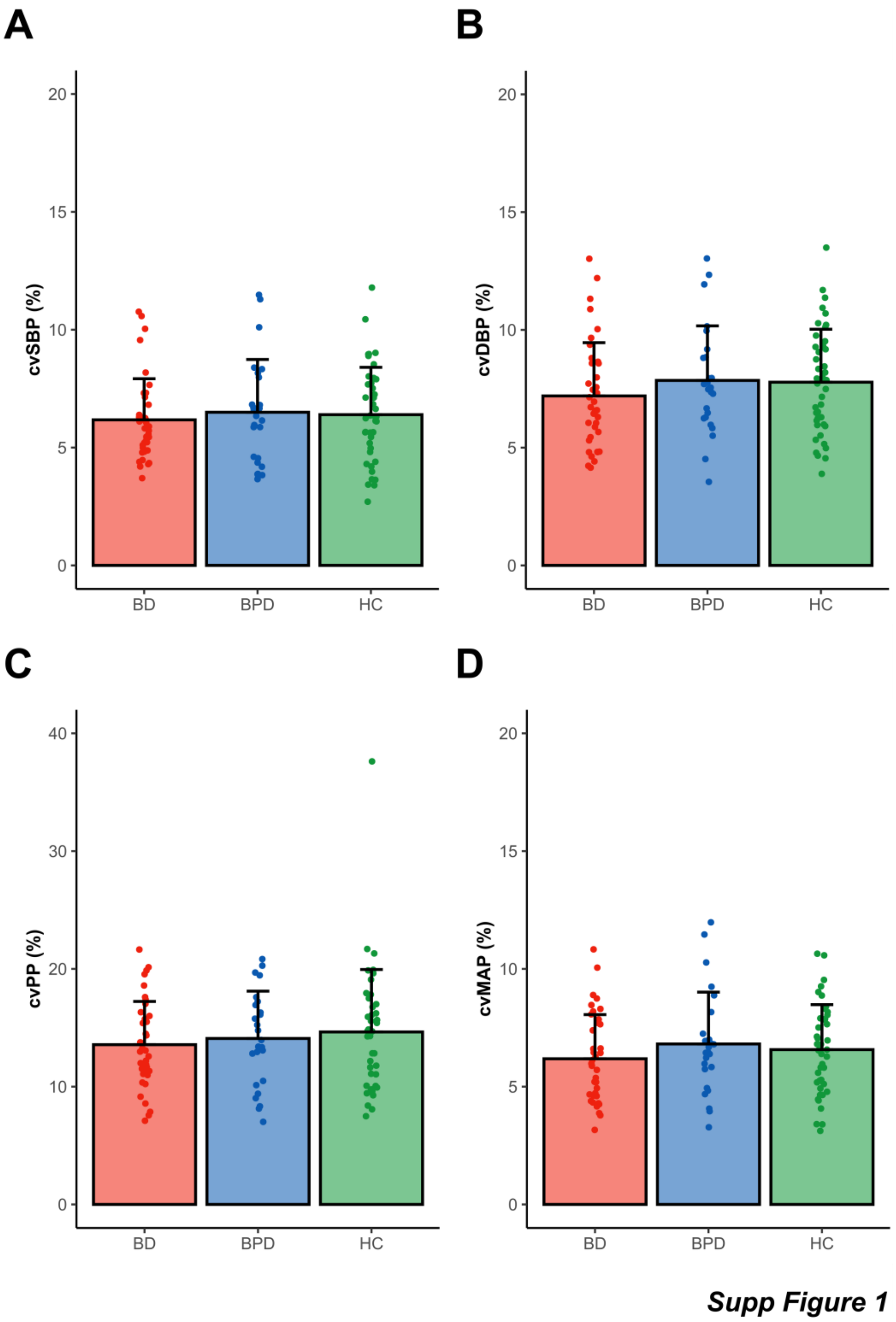

Supplementary Figure 1: Group-wise comparison of HBPM blood pressure variability

Bars represent group blood pressure variability estimated by the coefficient of variation of the week-long recording period with error bars indicating standard deviation. Variables plotted are as follows (A) systolic blood pressure (SBP); (B) diastolic blood pressure (DBP); (C) pulse pressure (PP); (D) mean arterial pressure (MAP). All comparisons control for age and BMI as covariates with no significant differences detected between BD, BPD and HC.

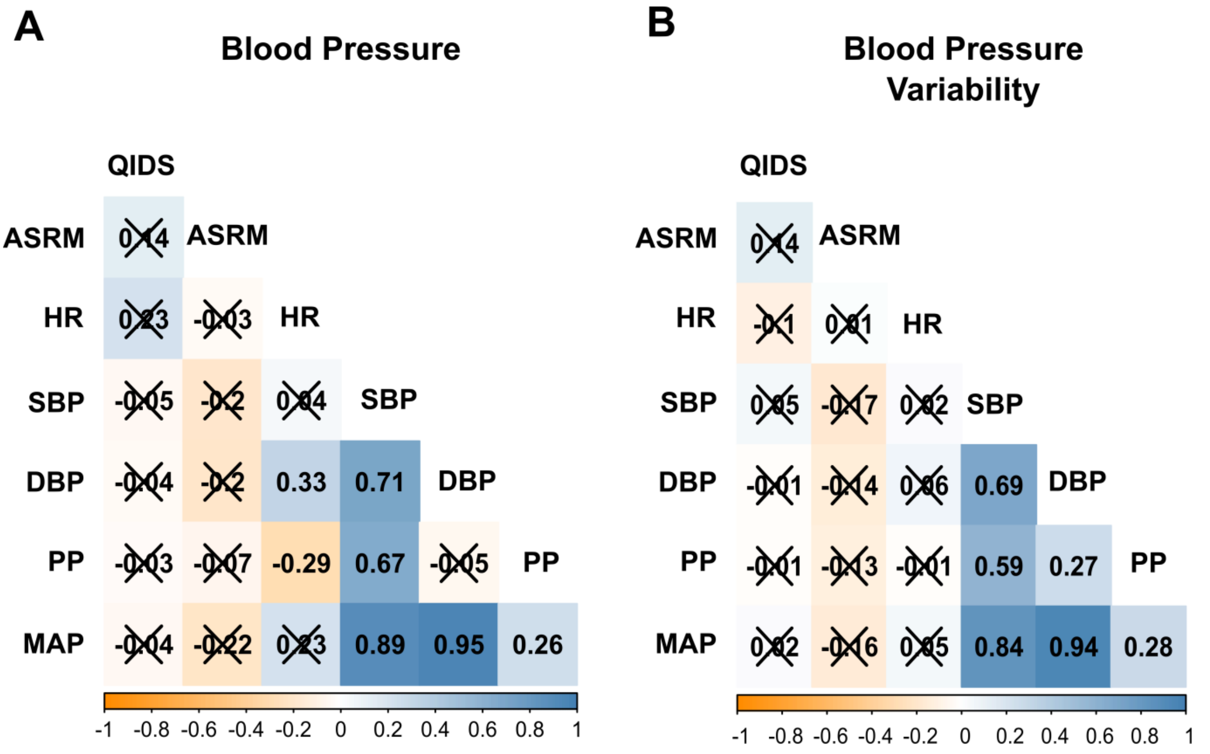

Supp Figure 2

Supplementary Figure 2: Correlation between depressive and manic symptomatology with mean blood pressure and blood pressure variability

Correlation plot shows Spearman rank-order correlation matrix of depressive symptoms (QIDS score) and manic symptoms (ASRM score) and their association with (A) mean BP variables and (B) blood pressure variability measured via the coefficient of variation. QIDS = quick inventory of depressive symptomatology; ASRM = Altman self-rating mania scale. Strength and direction of correlation is indicated by colour legend. Crossed-out digits represent non-significant correlations (P>0.05, FDR correction applied).

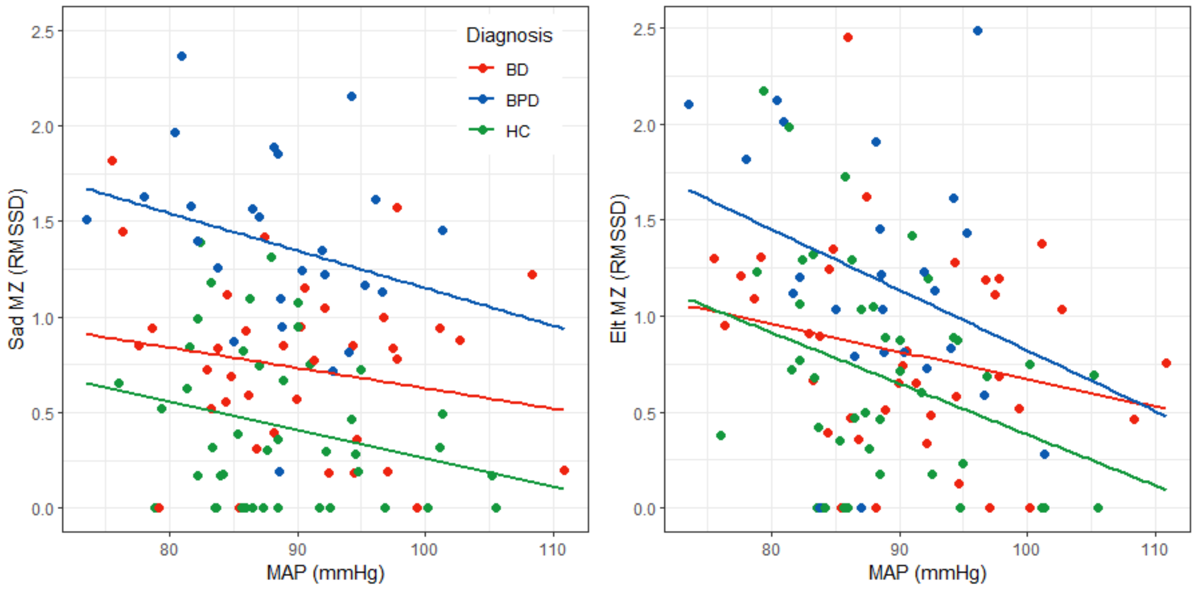

Supp Figure 3

Supplementary Figure 3: Association between mood instability and blood pressure segregated by group

Scatter plot indicating the inverse relation between mood instability and blood pressure for each group. Example plots show similar negative association with mean blood pressure for negative mood (example 'Sad') as for positive mood (example 'Elated') for BD, BPD and HC.
